# National Institutes of Health Funding to Support Radiation Oncology Research: A Comparative Trend Analysis Over a Decade, 2011-2021

**DOI:** 10.1016/j.adro.2025.101767

**Published:** 2025-04-22

**Authors:** Amir Razavi, Michael K. Rooney, Clifton D. Fuller, James B. Yu, Neil T. Pfister, Charles R. Thomas, John M. Buatti, Sophia C. Kamran, Heather M. McGee, Debra Nana Yeboa, Ana P. Kiess, Andrew M. Baschnagel, Randall J. Kimple

**Affiliations:** a Department of Radiation Oncology, MD Anderson Cancer Center, Houston, Texas; b Department of Radiation Oncology, St. Francis Hospital, Hartford, Connecticut; c Department of Radiation Oncology, University of Alabama at Birmingham School of Medicine, Birmingham, Alabama; d Radiation Oncology Department, Dartmouth Cancer Center and Geisel School of Medicine, Lebanon, New Hampshire; e Department of Radiation Oncology, Holden Comprehensive Cancer Center, University of Iowa, Iowa City, Iowa; f Department of Radiation Oncology, Massachusetts General Hospital Cancer Center, Harvard Medical School, Boston, Massachusetts; g Department of Radiation Oncology and Immuno-Oncology, City of Hope, Duarte, California; h Department of Radiation Oncology and Molecular Radiation Sciences, The Johns Hopkins University School of Medicine, Baltimore, Maryland; i Department of Human Oncology, UW Carbone Cancer Center, University of Wisconsin School of Medicine and Public Health, Madison, Wisconsin

## Abstract

**Purpose:**

Funding to support radiation oncology discovery and research is essential for advancement in therapeutic strategies to improve outcomes for patients with cancer. We aimed to comprehensively characterize trends in National Institutes of Health (NIH) funding that supports radiation oncology research over time to identify trends, successes, and areas for improvement.

**Methods and Materials:**

We queried the NIH Research Portfolio Online Reporting Tools Expenditures and Results database to identify all awarded grants to support radiation oncology research conducted by principal investigators at academic centers, using 3 individual years as representative samples (2011, 2016, and 2021). Abstracts and keywords for resulting grants were manually searched to identify resulting awards topically related to the field of radiation oncology; principal investigators departmental affiliation was also used as a supplemental method serving as a sensitivity analysis to define radiation oncology-related research. Descriptive statistics were used to describe patterns in funding. χ^2^ testing was used to assess differences in proportions of categorical variables.

**Results:**

Less than 0.5% of the total NIH budget and < 2% of the total National Cancer Institute budget supported radiation oncology research during the representative study years. There were no significant changes in this allocation pattern over time. A small cohort of institutions held a relatively large proportion of NIH-supported radiation oncology grant funding. Individuals holding PhDs alone received the majority of funding (62%), whereas those with dual-degrees (MD/PhD) held 21% of funding, and those with MD alone were awarded 17% of funding. There was a trend toward an increased proportion of grants awarded to MD/PhDs over time (24% vs 15% in 2021 and 2011, respectively, *P* = .075).

**Conclusions:**

Despite radiation therapy's essential role in multidisciplinary cancer care, NIH, and National Cancer Institute funding to support radiation oncology research has remained disproportionally low over the last decade. These data may be useful to inform future policy aimed at promoting research advancement in radiation oncology both at the micro (individual) as well as macro (institutional and national) level.

## Introduction

Radiation therapy has been a central component of cancer care for over 100 years and remains a cornerstone of modern multimodality cancer treatment. Indeed, approximately 50% of patients with cancer receive radiation therapy at some point during their disease course.^1^ Despite this clear importance, however, only a minority (5.3%) of recent cancer clinical trials used radiation therapy as an experimental intervention,^2^ suggesting that advancements in radiation therapy are moving at a slower pace compared with other modalities, particularly systemic agents.

Although this trend is multifactorial, disproportionate research funding allocation is one potential contributing factor.^3^ Institutional financial resources can be a prohibitive barrier for conducting cutting-edge basic, translational, or clinical research and therefore external financial support to mitigate these barriers is often required.^4^ As an agency of the United States Department of Health and Human Services, the National Institutes of Health (NIH) is the foremost medical research foundation aimed at expanding knowledge in medical sciences to advance the country's health at large. The National Cancer Institute (NCI) is one component of the NIH and receives approximately 15% of the NIH budget as the government's principal agency for advancing cancer research.

This investigation was initiated by a group concerned about development of research-focused investigators in the field of radiation oncology and aimed to provide a longitudinal characterization of the state of NIH- and NCI-supported research in the field from 2011 to 2021. We hypothesized that funding dollars for radiation oncology research would be disproportionately low compared with radiation therapy's clinical use and that funding trends would not improve over time. Further, we sought to identify whether particular institutions obtained large proportions of funding, and whether institutional trends were persistent over time. These results not only could be used to inform national-level policies regarding funding allocation but also aid in defining research priorities at the institutional level.

## Methods and Materials

This study adopted rigorous methodology for evaluating the NIH funding landscape for radiation oncology, using data from the NIH Research Portfolio Online Reporting Tools Expenditures and Results (RePORTER), which are a publicly available database that contains a collection of all NIH-funded projects and grants.^5^ We first identified all grants under the NIH's “Radiation – Diagnostic/Oncology” department categorization. Because this departmental categorization includes both diagnostic radiology and radiation oncology, we assigned topical assignments of “radiation oncology” versus “other” to identify the final cohort of grants to be included for analysis. Our approach allowed us to evaluate funding chronologically from 2011, 2016, and 2021. A supplementary review using different methodology was done as a sensitivity analysis to ensure data integrity, reproducibility, and robustness.

### Methodology

**Figure 1:**
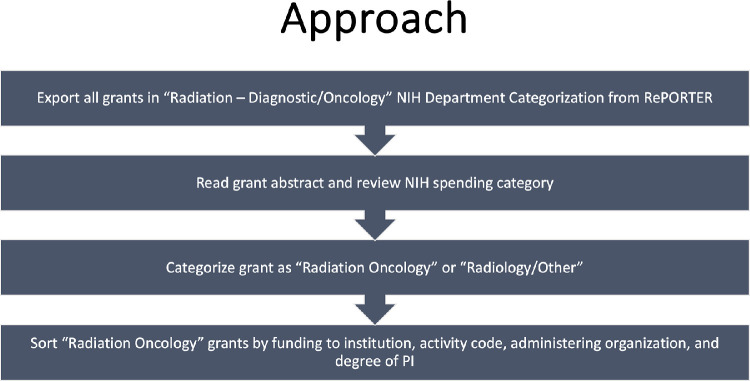
Methodology to designate grant as radiation oncology-related versus other. *Abbreviations:* NIH = National Institutes of Health; PI = principal investigator.

We began by identifying fiscal year (FY) 2021 grants within the RePORTERs “Radiation – Diagnostic/Oncology” NIH department categorization. The categorization of grants as “Radiation Oncology” or “Radiology/Non-Radiation Oncology” was determined manually by reviewing the content of the grant abstracts and tagged spending categories, as depicted in Fig. 1. It is important to note that some institutions, such as the Harvard Radiation Oncology Program, Memorial Sloan Kettering Cancer Center, Mayo Clinic, and City of Hope, among others, do not have recipient departments such as “Radiation Oncology” that are identified by the NIH. Therefore, grants awarded to radiation oncology investigators at these institutions did not appear within our initial search. For these grants, we used the NIH spending category tag “radiation oncology.” The grants with the “radiation oncology” spending category tag were then manually reviewed and coded as “radiation oncology” or “other” based on the details of the grant abstract. To ensure all academic radiation oncology programs were included, we used the Accreditation Council for Graduate Medical Education (ACGME) Radiation Oncology program list as an additional check and cross-referenced whether they had a recipient “Radiation Oncology” department within the NIH RePORTER.

The entirety of the methodology was not collected using the “radiation oncology” spending category because the absolute number of grants is underreported if that spending category is the sole category used. For this reason, it is important to note that some of the omitted institutions may be underreported on their radiation oncology funding because they were not all tied to a department of radiation oncology. Coding for this approach was performed by one study author (AR) and an audit of a random 10% sample of the data was performed by another study author (MKR). There was high agreement between coders (96% agreement, κ 0.91). This ensured the incorporation of all pertinent institutions, including those not captured within the initial search by department categorization.

We examined changes and trends in funding allocation over a decade with a focus on 3 years: 2011, 2016, and 2021. The supplementary sensitivity analysis only covers 2021 as the methodology used made retrospective data collection difficult, as institutional affiliations may change over time.

### Supplementary approach (sensitivity analysis)

To ensure the quality of our methodology given the subjectiveness involved, a sensitivity analysis using a more objective approach was conducted. This sensitivity analysis started with the same initial search of the “Radiation – Diagnostic/Oncology” NIH department categorization. The main criterion for sorting the grants this time was the Principal Investigator's (PI) department appointment, which was manually retrieved from the PIs institutional website. This approach is detailed in Fig. E1. All data in this analysis were derived from the NIH RePORTER and institutional websites, so the accuracy of all tables and conclusions is contingent on the completeness of these data sources. It is important to note the omitted institutions were not included in the sensitivity analysis because of the lack of a recipient radiation oncology department. For this reason, some institutions who received NIH funding for radiation oncology research (or lead by radiation oncology faculty) may not be accurately assessed within the sensitivity analysis.

### Grant-level data and analysis

After identifying the final cohort of grants for analysis, we tabulated a variety of grant characteristics including PI institutional affiliation, PI degree, grant type/activity code, and administering organization. We used descriptive statistics to describe patterns in funding across groups. χ^2^ testing was used to assess differences in proportions of categorical variables. Statistical significance was defined as *P* < .05.

## Results

### National-level trends (NIH and NCI)

**Figure 2:**
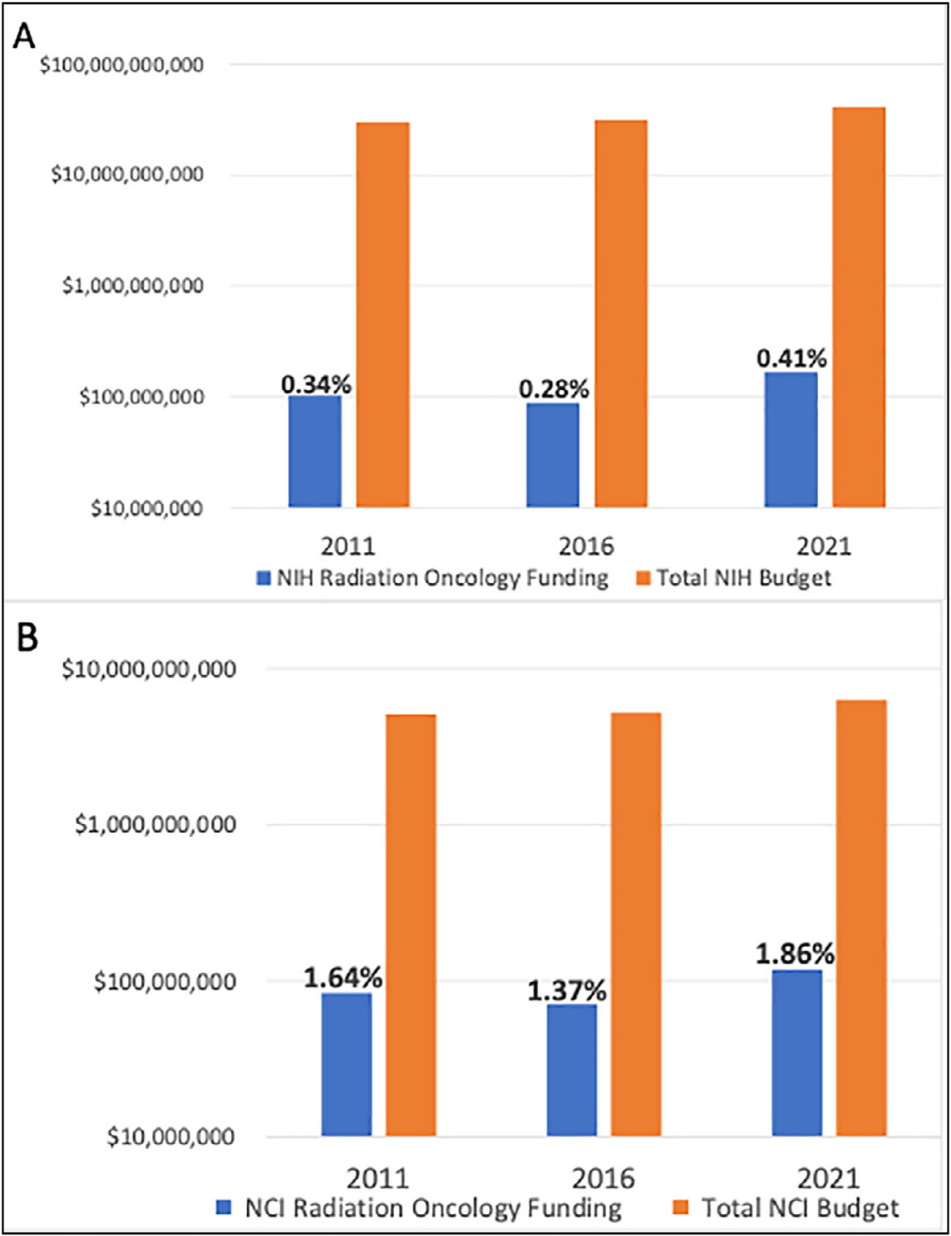
NIH (A) and NCI (B) funding to support radiation oncology compared with total budget across 2011, 2016, and 2021. Note the logarithmic scale to improve visualization. *Abbreviations:* NCI = National Cancer Institute; NIH = National Institutes of Health.

The NIH had a total budget of $40,886,000,000 in the 2021 FY, roughly 25% larger than the 2011 and 2016 NIH budgets totaling $29,936,000,000 and $31,486,000,000, respectively.^6^ Figure 2 shows trends in radiation oncology funding representation during the last decade based on 3 representative years during that period (2011, 2016, and 2021). Less than 0.5% of the total NIH budget supported radiation oncology research at academic institutions during this time.

The NCI budget similarly grew from $5,100,000,000 and $5,210,000,000 in 2011 and 2016 to $6,360,000,000 in 2021.^7^ The total amount of funding to radiation oncology also increased proportionally from 2011 to 2021, but the overall percentage of the NCI budget awarded to radiation oncology remained under 2% (Fig. 2B).

**Figure 3:**
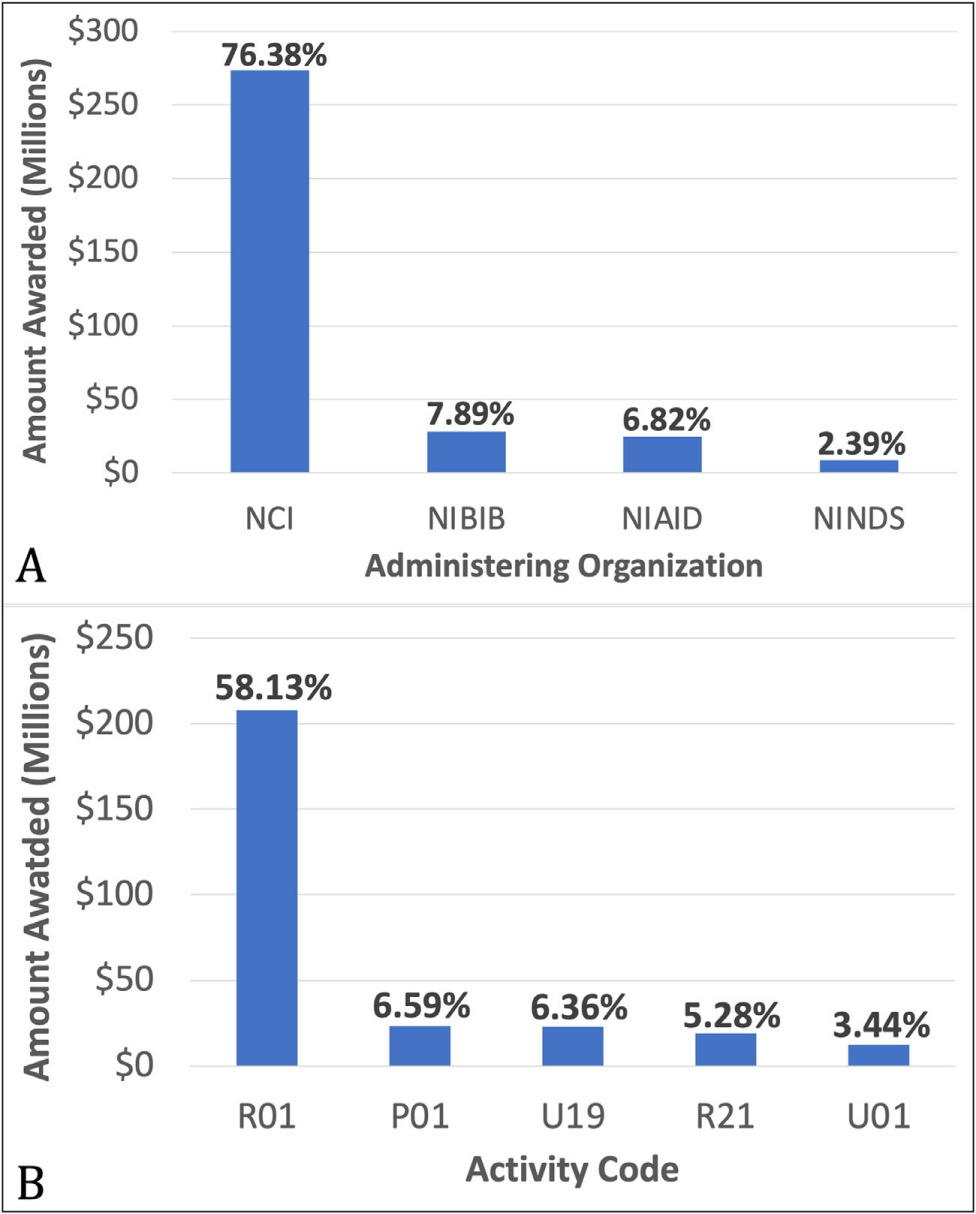
Top NIH administering institutions (A) and funded activity codes (B) to radiation oncology across 2011, 2016, and 2021. *Abbreviations:* NCI = National Cancer Institute; NIH = National Institutes of Health, NIBIB = National Institute of Biomedical Imaging and Bioengineering, NIAID = National Institute of Allergy and Infectious Diseases, NINDS = National Institute of Neurological Disorders and Stroke.

Distributions of funding according to administering organization are summarized in Fig. 3A. A total of 682 radiation oncology grants were issued by the NCI during 2011, 2016, and 2021 and totaled $273,449,029, composing 76.38% of the $358,030,070 total amount awarded to radiation oncology departments by the NIH. Collectively, 18 administering organizations awarded the 864 total grants, as shown in Table E1. The top administering organizations by amount awarded are shown in Fig. 3A. Distributions of grants by activity code are summarized in Fig. 3B. The most common grant type was R01s (research project grants), comprising 503 awards that represented 58.13% of all radiation oncology grant funds. A total of 43 activity codes were funded. A full description is provided in Table E2. Figure 3B shows the top funded NIH activity codes for radiation oncology grants across 2011, 2016, and 2021.

**Figure 4:**
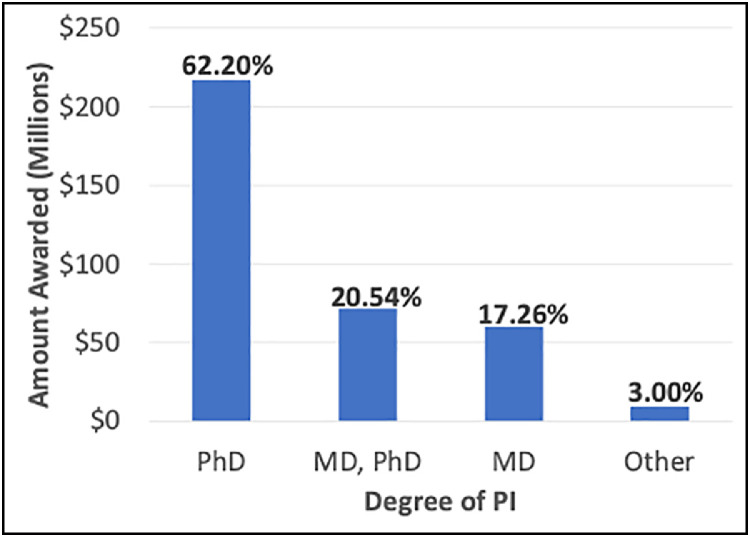
Radiation oncology funding by degree of PI from 2011, 2016, and 2021 combined. *Abbreviation:* PI = principal investigator.

PI characteristics including primary degree were analyzed and are summarized in Fig. 4. A total of 62.20% of grants were awarded to PhDs, whereas dual-degree MD/PhDs and MDs received 20.54% and 17.26% of funding, respectively. Proportions of awards by PI degree over the study period were also assessed and although not statistically significant, the percentage of grants awarded to dual-degree MD/PhDs rose throughout the course of the decade (24% vs 15% in 2021 and 2011, respectively, *P* = .075).

Total funding was $598,088,008, encompassing 1145 grants within the NIHs departmental “Radiation – Diagnostic/Oncology” categorization in FY 2021, of which 301 were awarded to radiation oncology departments. Funds totaled $121,520,769, comprising 20.32% of the total funding to the category (Fig. E2).

### Institutional-level trends

The NIH RePORTER yielded 1145 grants from the 2021 FY for the “Radiation – Diagnostic/Oncology” department tag. Of those 1145 grants, 301 were identified as supporting radiation oncology research. The remainder of the grants supported diagnostic radiology or other research. An additional 90 radiation oncology grants were identified from initially omitted institutions. The total 391 grants awarded to radiation oncology in 2021 were awarded across 61 institutions. FY 2016 had a drop in the number of grants awarded with 860 total awarded to the “Radiation – Diagnostic/Oncology” tag with 150 of the 860 being identified as radiation oncology. An additional 54 radiation oncology grants were identified from initially omitted institutions. In total, 2016 had 204 radiation oncology grants awarded across 50 different institutions. For 2011, 219 of 1217 total grants from the category were deemed to be radiation oncology supporting grants. Forty-one additional grants were awarded to institutions that did not appear in the initial search, bringing the total for 2011 to 260 total grants awarded across 54 different institutions. A complete list of institutions receiving NIH-supported radiation oncology funding during the most recent collected year, FY 2021, is shown in Tables E3 and E4. Table 1 provides a snapshot of the top 10 funded institutions in radiation oncology over the course of 2011, 2016, and 2021.

**Table 1:** Top 10 funded institutions from National Institutes of Health (NIH) to support radiation oncology research from 2011, 2016, and 2021

2011				
Ranking	Institution	2011 radiation oncology funding amount	2011 total funding	Radiation oncology as % of total funding
**1**	Sloan Kettering Institute for Cancer Research	$9,530,287	$109,147,785	8.73%
**2**	Massachusetts General Hospital	$8,647,896	$343,883,433	2.51%
**3**	Stanford University	$8,308,524	$332,383,758	2.50%
**4**	University of Michigan at Ann Arbor	$7,481,824	$459,413,187	1.63%
**5**	Johns Hopkins University	$6,811,308	$625,134,944	1.09%
**6**	Washington University	$5,288,588	$371,213,244	1.42%
**7**	University of Texas MD Anderson Cancer Center	$4,713,450	$152,299,097	3.09%
**8**	University Of California, Los Angeles	$4,517,591	$356,782,261	1.27%
**9**	Virginia Commonwealth University	$3,893,498	$79,227,663	4.91%
**10**	University of Pennsylvania	$2,656,795	$462,600,262	0.57%

2016				
Ranking	Institution	2016 radiation oncology funding	Institution total 2016 NIH funding	% of grant monies awarded to radiation oncology
**1**	Sloan Kettering Institute for Cancer Research	$8,376,636	$132,376,439	6.33%
**2**	Massachusetts General Hospital	$8,185,643	$364,981,379	2.24%
**3**	Columbia University Health Sciences	$7,583,702	$381,647,091	1.99%
**4**	University of Pennsylvania	$5,751,744	$478,866,008	1.20%
**5**	University of Michigan at Ann Arbor	$5,219,714	$486,690,808	1.07%
**6**	Stanford University	$5,035,505	$427,012,784	1.18%
**7**	University of California, San Francisco	$4,647,740	$577,576,919	0.80%
**8**	University of Pittsburgh at Pittsburgh	$4,142,379	$475,851,374	0.87%
**9**	University of Texas MD Anderson Cancer Center	$3,906,773	$124,463,282	3.14%
**10**	Duke University	$3,033,034	$416,881,431	0.73%

2021				
Ranking	Institution	2021 radiation oncology funding amount	Institution total 2021 NIH funding	% of grant monies awarded to radiation oncology
**1**	Sloan Kettering Institute for Cancer Research	$10,633,398	$186,945,694	5.69%
**2**	University of Texas MD Anderson Cancer Center	$9,826,748	$172,556,179	5.69%
**3**	Massachusetts General Hospital	$9,480,779	$600,667,106	1.58%
**4**	Johns Hopkins University	$8,349,004	$824,856,274	1.01%
**5**	Stanford University	$8,131,174	$611,354,637	1.33%
**6**	University of California, San Francisco	$7,656,147	$709,018,244	1.08%
**7**	Columbia University Health Sciences	$7,297,958	$580,097,026	1.26%
**8**	University of Pennsylvania	$6,126,984	$641,789,096	0.95%
**9**	University of Michigan at Ann Arbor	$5,353,137	$609,038,367	0.88%
**10**	Washington University	$5,302,089	$623,444,643	0.85%

Of 61 institutions receiving radiation oncology funding in 2021, 38 institutions received < 1% of their respective institutions’ total 2021 NIH funding. Three institutions in our sample, each a cancer focused institution, Sloan Kettering Institute, MD Anderson Cancer Center, and City of Hope/Beckman Research Institute had an NIH award pool for which radiation oncology funding represented approximately 5% of the full pool (5.7%, 5.7%, and 4.8%, respectively).

## Discussion

This observational study aimed to rigorously examine the current state of NIH-supported radiation oncology research funding over the last decade. We found that < 2% of the total funding from the NCI and < 0.5% from the NIH has been allocated to support radiation oncology research during this time, a trend which has not changed over the study period. Further, a small group of institutions tend to obtain a large proportion of radiation oncology funding. Most funding was awarded to PIs holding PhDs and individuals with MDs alone were a minority of awardees. These results can be used not only to advocate for greater governmental support for radiation oncology research but also can be used by institutional leadership to guide policy and achieve institutional-level missions.

Prior research on the state of funding in radiation oncology has highlighted the need for increased support for radiation oncology research by governmental agencies.^8^ A 2017 American Society for Radiation Oncology (ASTRO) analysis called for a reversal of the decreased amount of funding to radiation oncology during a time when the field was experiencing heightened importance to cancer care.^9^ A similar study by Steinberg et al^3^ noted many findings that raised “great concern” regarding the state of NIH funding in radiation oncology. These findings included < 0.3% of NIH funding going to radiation oncology PIs, and 1.6% of cancer research funding supporting radiation oncology-related investigation. Our study seeks to build on these prior studies and survey the current state of funding in radiation oncology. Importantly, our analysis expanded to a more current era up to the year 2021 using multiple methods of data collection to ensure data integrity. As such, these results will thus provide a contemporary and holistic description of the shifting landscape of radiation oncology research funding.

The findings from this study largely corroborate prior research in this space and continue to show a stark contrast between the use of radiation therapy in cancer treatment and the proportion of funding dedicated to its research.^10^ Adequate research funding is imperative to advancing our understanding of radiation therapy, improving treatment protocols, and ultimately enhancing patient outcomes.^11^

The underlying etiology behind this trend is multifaceted and may include a lack of awareness among funding bodies and researchers alike regarding the integral role of radiation therapy in cancer treatment.^12^ Furthermore, there could be disproportionate representation among leadership positions within funding agencies that could bias funding allocation.^13^ To address this issue, a concerted effort is required from the radiation oncology community, including curriculum reform to include grant writing primers and clinical trial design primers as a normative component of postgraduate training. Increased advocacy and awareness campaigns aimed at highlighting the importance of radiation therapy in cancer management and the need for proportionate research funding are crucial.^14^

An encouraging trend that emerged in our data are the increased number of dual-degree MD-PhD recipients receiving grants from the NIH, particularly in the last study year, 2021. Data from Association of American Medical Colleges (AAMC) shows that in 2021, 13.2% of all radiation oncology residents were MD/PhDs, which is significantly higher than the 3.3% across all specialties combined. In recent years, there has been significant concern within the field that despite strong recruitment of dual-degree students into radiation oncology, there has been significant loss of research talent, with lack of funding often being cited as a large barrier to continued pursuit of a physician-scientist career.^15^ Although our data certainly do suggest that radiation oncology in general is underfunded, it is encouraging that an increasing proportion of grant awardees in the field were from a dual-degree background. MD/PhD researchers bring a unique perspective to the field, bridging the gap between patient care and laboratory research, and therefore represent a key population to drive innovation in the field.^16^ Provision of adequate startup packages, protected research time, and high-quality mentoring are important components to support early-career researchers and enable them to generate the necessary preliminary data to successfully compete for peer reviewed NIH funding. Continued support for these investigators from the federal government will not only be critical for growth of radiation oncology as a field but more importantly encourage discovery to ultimately improve lives for patients with cancer.

An unintended but important finding from this investigation was the difficulty in accurately categorizing radiation oncology research using the current RePORTER interface, which ultimately necessitated multiple methods of data collection to ensure data accuracy. One critical issue is a lack of a standalone “Radiation Oncology” department categorization within the NIH RePORTER system. Currently, radiation oncology is often combined with diagnostic radiology under the “Radiation – Diagnostic/Oncology” category. This amalgamation not only dilutes the visibility of radiation oncology but also complicates the process of accessing and assessing the true state of funding allocated specifically to radiation oncology research and discriminating it from radiology research. Moreover, authors and grant writers must be diligent in correctly labeling awarded grants as “Radiation Oncology” when appropriate. Proper categorization at the authorial level will enable a more systematic and efficient tracking of research outputs and their corresponding funding sources, thereby fostering transparency and accountability in the allocation and use of research funds. We strongly advocate for a formal annotation method in grant reporting to flag research efforts as radiation oncology-related. This is long overdue.

Despite its strength of a rigorous data collection methodology, this study has several limitations. First, the NIH department categorization does not include certain institutions as recipient “Radiation Oncology” programs, which ultimately necessitated an alternative data collection method serving as a sensitivity analysis. However, our methodology itself may be prone to personalized bias as grants were manually coded by topic and thus results may not be entirely reproducible. Encouragingly, however, results by our primary data collection and the supplementary sensitivity analysis yielded the same general conclusions. Additionally, a random audit supported the accuracy of manual data collection. Second, within our sensitivity analysis, PIs might have multiple department appointments within their institution. In these cases, radiation oncology was chosen as the default, which may inadvertently yield inaccurately higher estimates of funding by this analysis. Third, this study excludes any funding from other agencies and organizations (eg, cooperative groups and private organizations). Finally, grants with multiple PIs might have been missed if the primary PI did not have a radiation oncology appointment but would have otherwise met inclusion criteria.

This project was begun by a professional committee but does not represent an official work-product of this committee. Given the findings observed, the authors recommend the following steps be undertaken to improve engagement of stakeholders:

1. Recommendation that the NIH separates radiation oncology from diagnostic radiology in the department categorization in the NIH RePORTER so we can accurately track funding.
2. Recommendation that ASTRO and the Society of Chairs of Academic Radiation Oncology Programs undertake a formal Request for Information/survey to identify barriers to entry or bars to continuation of radiation research across the career continuum.
3. Recommendation for additional support for research time protection and early-career NIH awards for early to middle career faculty through ASTRO and the Society of Chairs of Academic Radiation Oncology Programs.
4. Recommendation that NIH engage radiation oncologists as referees across Center for Scientific Review study sections to ensure that all applications have relevant referee expertise.
5. Invite the NCI Director to make an annual presentation to the ASTRO meeting, similar to that which occurs at the annual American Associate for Cancer Research (AACR) and American Society of Clinical Oncology (ASCO) meetings.

In conclusion, our study highlights a clear and concerning disparity between the use of radiation therapy in cancer treatment and the amount of funding allocated to radiation oncology research by the NIH and NCI. Addressing this imbalance is essential to advancing the field of radiation oncology, improving treatment outcomes, and ultimately benefiting the multitude of patients with cancer who rely on radiation therapy as part of their treatment regimen.

## Disclosures

Clifton D. Fuller reports unrelated travel, honoraria, and speaker fee provision by National Institute of Health, Elekta AB, GE Healthcare, Varian/Siemens Healthineers, Philips Medical Systems, Australian & New Zealand Head and Neck Society, and University of Wisconsin Comprehensive Cancer Center; unrelated patent licensing royalties from Kallisio, Inc; received support for attending meetings and/or travel from the American Society for Radiation Oncology, European Society for Radiotherapy and Oncology, Australian & New Zealand Head and Neck Society, American Association for Physics in Medicine, and Radiological Society of North America; and serves on committees for the American Society for Radiation Oncology, American Society for Clinical Oncology, American Association for Physics in Medicine, and National Institute of Health. James B. Yu reports received grant from Pfizer and Myovant; consulting fees from Boston Scientific; payment from Myovant; and support for attending meetings and/or travel for STIC. Neil T. Pfister reports payment received from CDMRP/GDIT; and participation in advisory board and equity from Numenos. John M. Buatti reports participation in an advisory board for ASTRO. Sophia C. Kamran reports receipt of the Prostate Cancer Foundation Young Investigator Award and DOD Prostate Cancer Idea Development Award (HT9425-24-1-0655). Heather M. McGee reports grants received from National Institutes of Health. Debra Nana Yeboa reports grants received from Brockman Medical Research, Robert Wood Johnson, and MD Anderson Cancer Center; and holds leadership or fiduciary roles in boards, societies, committees, or advocacy groups (paid or unpaid) with the Practical Radiation Oncology Editorial Board, ASTRO Science Council, and SNO Radiation Track Sciences. Ana P. Kiess reports travel support and participation in uncompensated advisory boards from Novartis. Andrew M. Baschnagel reports grants received from University of Wisconsin Comprehensive Cancer Center. Randall J. Kimple reports consulting fees received from HunaTek and Guidepoint Global and research funding paid to the University of Wisconsin from the NIH, Oncohost, and Merck.

## Appendix Supplementary materials

Manuscript Supplementary Image, application 1

## Footnotes

Supplementary material associated with this article can be found in the online version at doi:10.1016/j.adro.2025.101767.
