# Supplementary material for: National Institutes of Health Funding to Support Radiation Oncology Research: A Comparative Trend Analysis Over a Decade, 2011-2021
Source: Adv Radiat Oncol. 2025 Apr 22;10(6):101767. doi: 10.1016/j.adro.2025.101767 (PMC12051116; doi:10.1016/j.adro.2025.101767)

Supplemental tables and figures:

Supplemental figure 1: Sensitivity analysis approach

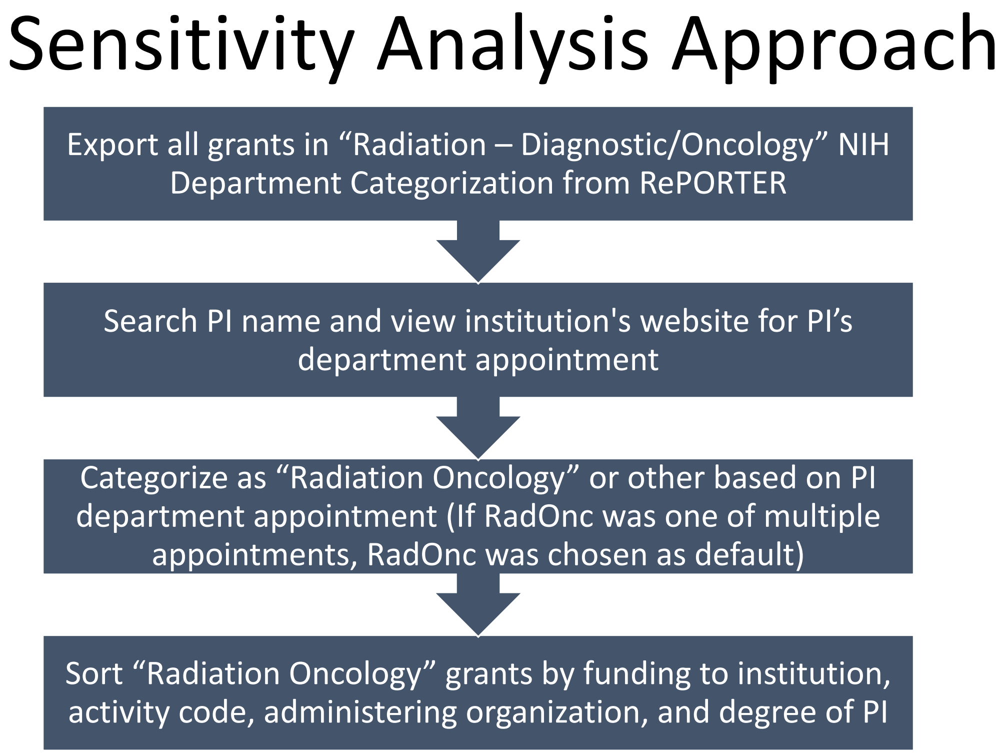

Supplemental table 1: Awards to radiation oncology by NIH administering organization across 2011, 2016, and 2021

Administering Organization	Number of Grants	Amount	% of Total
National Cancer Institute (NCI)	682	$273,449,029.00	76.38%

National Institute of Biomedical Imaging and Bioengineering (NIBIB)	73	$28,237,045.00	7.89%
National Institute of Allergy and Infectious Diseases (NIAID)	14	$24,422,593.00	6.82%
National Institute of Neurological Disorders and Stroke (NINDS)	23	$8,550,998.00	2.39%
Office of the Director (OD)	13	$5,719,154.00	1.60%
National Institute of Dental and Craniofacial Research (NIDCR)	15	$4,376,900.00	1.22%
National Institute of Environmental Health Sciences (NIEHS)	9	$2,854,916.00	0.80%
National Heart, Lung, and Blood Institute (NHLBI)	6	$3,177,058.00	0.89%
Agency for Healthcare Research and Quality (AHRQ)	7	$2,106,302.00	0.59%
National Institute of General Medical Sciences (NIGMS)	6	$1,562,112.00	0.44%
National Institute on Aging (NIA)	4	$1,304,735.00	0.36%
National Center for Research Resources (NCRR)	5	$816,779.00	0.23%
National Institute of Child Health and Human Development (NICHD)	1	$493,296.00	0.14%
National Library of Medicine (NLM)	1	$461,828.00	0.13%

National Center for Chronic Disease Prevention and Health Promotion (NCCDPHP)	1	$260,242.00	0.07%
Total	864	$358,030,070.00	100.00%

Supplemental table 2: Awards to radiation oncology by NIH activity code across 2011, 2016, and 2021

Activity Code	Number of Grants	Total Funding	% of Total
R01 (Research Project)	503	$208,130,104.00	58.13%
P01 (Research Program Projects)	30	$23,593,996.00	6.59%
U19 (Cooperative Agreements)	13	$22,765,168.00	6.36%
R21 (Exploratory/Developmental Grants)	86	$18,887,268.00	5.28%
U01 (Cooperative Agreements)	22	$12,304,513.00	3.44%
R35 (Outstanding Investigator Award)	10	$8,018,998.00	2.24%
R37 (Method to Extend Research in Time Award - MERIT)	15	$6,691,084.00	1.87%
T32 (Institutional National Research Service Award)	19	$4,736,850.00	1.32%
P50 (Specialized Center)	7	$4,900,733.00	1.37%

DP5 (Early Independence Award)	11	$4,519,154.00	1.26%
U24 (Resource - Related Research Projects - Cooperative Agreements)	5	$4,446,244.00	1.24%
K12 (Physician Scientist Award [Program] - PSA)	8	$4,702,469.00	1.31%
K08 (Clinical Investigator Award - CIA)	22	$4,381,270.00	1.22%
U10 (Cooperative Clinical Research - Cooperative Agreements)	4	$4,807,619.00	1.34%
P41 (Biotechnology Resource Grants)	6	$3,345,616.00	0.93%
R25 (Education Projects)	10	$2,791,943.00	0.78%
UG1 (Clinical Research Cooperative Agreements - Single Project)	3	$2,527,147.00	0.71%
S10 (Biomedical Research Support Shared Instrumentation Grants)	4	$2,016,779.00	0.56%
UH3 (Exploratory/Developmental Cooperative Agreement Phase II)	3	$1,345,147.00	0.38%
U54 (Specialized Center - Cooperative Agreements)	3	$1,743,921.00	0.49%
P30 (Center Core Grants)	6	$1,282,190.00	0.36%
R00 (Research Transition Award)	4	$967,115.00	0.27%
R18 (Research Demonstration and Dissemination Projects)	2	$772,873.00	0.22%
R03 (Small Research Grants)	13	$1,081,623.00	0.30%
K99 (Career Transition Award)	5	$ 728,077.00	0.20%

UG3 (Phase 1 Exploratory/Developmental Cooperative Agreement)	3	$1,735,008.00	0.48%
K07 (Academic/Teacher Award - ATA)	4	$581,510.00	0.16%
K22 (Career Transition Award)	3	$507,825.00	0.14%
R50 (Research Specialist Award)	3	$426,347.00	0.12%
N01 (R&D Contracts)	1	$377,050.00	0.11%
N01 (R&D Contracts)	1	$377,050.00	0.11%
K01 (Research Scientist Development Award - Research & Training)	4	$534,769.00	0.15%
F32 (Postdoctoral Individual National Research Service Award)	6	$291,967.00	0.08%
U58 (Chronic Disease Control Cooperative Agreement)	1	$260,242.00	0.07%
F31 (Predoctoral Individual National Research Service Award)	8	$294,999.00	0.08%
K76 (Emerging Leaders Career Development Award)	1	$242,584.00	0.07%
F30 (Individual Predoctoral NRSA for M.D./Ph.D Fellowships)	7	$292,231.00	0.08%
K05 (Research Scientist Award)	1	$164,278.00	0.05%
K25 (Mentored Quantitative Research Career Development)	1	$145,830.00	0.04%

K02 (Research Scientist Development Award - Research)	1	$100,562.00	0.03%
P20 (Exploratory Grants)	2	$99,641.00	0.03%
K00 (Post-doctoral Transition Award)	1	$88,776.00	0.02%
R13 (Conference)	2	$21,500.00	0.01%
Total	864	$358,030,070.00	100.00%

Supplemental table 3: 2021 funding by institution using the primary methodology.

Rad Onc Ranking	Institution	2021 Rad Onc Funding Amount	Institution Total 2021 NIH Funding	% of Grant Monies Awarded to Radiation Oncology
1	SLOAN-KETTERING INST CAN RESEARCH	$10,633,398.00	$186,945,694.00	5.69%
2	UNIVERSITY OF TX MD ANDERSON CAN CTR	$9,826,748.00	$172,556,179.00	5.69%
3	MASSACHUSETTS GENERAL HOSPITAL	$9,480,779.00	$600,667,106.00	1.58%

4	JOHNS HOPKINS UNIVERSITY	$8,349,004.00	$824,856,274.00	1.01%
5	STANFORD UNIVERSITY	$8,131,174.00	$611,354,637.00	1.33%
6	UNIVERSITY OF CALIFORNIA, SAN FRANCISCO	$7,656,147.00	$709,018,244.00	1.08%
7	COLUMBIA UNIVERSITY HEALTH SCIENCES	$7,297,958.00	$580,097,026.00	1.26%
8	UNIVERSITY OF PENNSYLVANIA	$6,126,984.00	$641,789,096.00	0.95%
9	UNIVERSITY OF MICHIGAN AT ANN ARBOR	$5,353,137.00	$609,038,367.00	0.88%
10	WASHINGTON UNIVERSITY	$5,302,089.00	$623,444,643.00	0.85%
11	UNIVERSITY OF WISCONSIN-MADISON	$5,094,338.00	$367,581,113.00	1.39%
12	WAKE FOREST UNIVERSITY HEALTH SCIENCES	$4,695,468.00	$123,142,161.00	3.81%
13	CASE WESTERN RESERVE UNIVERSITY	$4,516,453.00	$177,329,462.00	2.55%
14	UT SOUTHWESTERN MEDICAL CENTER	$4,162,140.00	$255,325,647.00	1.63%
15	BECKMAN RESEARCH INSTITUTE/CITY OF HOPE	$3,555,015.00	$74,329,548.00	4.78%

16	DANA-FARBER CANCER INST	$3,445,556.00	$159,777,039.00	2.16%
17	UNIVERSITY OF CALIFORNIA-IRVINE	$3,401,748.00	$172,170,439.00	1.98%
18	UNIVERSITY OF CALIFORNIA LOS ANGELES	$3,393,383.00	$590,125,984.00	0.58%
19	ICAHN SCHOOL OF MEDICINE AT MOUNT SINAI	$3,363,657.00	$441,276,662.00	0.76%
20	OHIO STATE UNIVERSITY	$3,212,889.00	$230,407,670.00	1.39%
21	UNIVERSITY OF PITTSBURGH AT PITTSBURGH	$3,164,096.00	$597,804,833.00	0.53%
22	YALE UNIVERSITY	$2,903,998.00	$557,452,201.00	0.52%
23	UNIVERSITY OF CALIFORNIA, SAN DIEGO	$2,832,871.00	$549,764,120.00	0.52%
24	MEDICAL COLLEGE OF WISCONSIN	$2,630,698.00	$115,235,521.00	2.28%
25	THOMAS JEFFERSON UNIVERSITY	$2,590,905.00	$81,449,194.00	3.18%
26	NEW YORK UNIVERSITY SCHOOL OF MEDICINE	$2,401,886.00	$809,311,644.00	0.30%
27	DUKE UNIVERSITY	$2,399,674.00	$731,237,450.00	0.33%

28	UNIVERSITY OF VIRGINIA	$2,099,952.00	$175,512,234.00	1.20%
29	EMORY UNIVERSITY	$2,097,859.00	$479,575,357.00	0.44%
30	MAYO CLINIC ROCHESTER	$1,979,784.00	$241,227,697.00	0.82%
31	NORTHWESTERN UNIVERSITY AT CHICAGO	$1,969,316.00	$367,542,487.00	0.54%
32	VANDERBILT UNIVERSITY MEDICAL CENTER	$1,873,589.00	$344,312,186.00	0.54%
33	UNIVERSITY OF CHICAGO	$1,738,652.00	$234,209,363.00	0.74%
34	UNIVERSITY OF UTAH	$1,720,655.00	$229,692,858.00	0.75%
35	UNIVERSITY OF WASHINGTON	$1,708,800.00	$543,508,259.00	0.31%
36	UNIVERSITY OF IOWA	$1,655,300.00	$183,267,904.00	0.90%
37	UNIVERSITY OF COLORADO DENVER	$1,533,690.00	$313,670,852.00	0.49%
38	UNIVERSITY OF ALABAMA AT BIRMINGHAM	$1,378,283.00	$327,424,300.00	0.42%
39	UNIVERSITY OF MARYLAND BALTIMORE	$1,171,933.00	$234,014,725.00	0.50%
40	UNIVERSITY OF TEXAS HLTH SCIENCE CENTER	$1,139,086.00	$95,847,459.00	1.19%
41	MEDICAL UNIVERSITY OF SOUTH CAROLINA	$1,089,127.00	$131,460,110.00	0.83%

42	BRIGHAM AND WOMEN'S HOSPITAL	$774,530.00	$357,068,821.00	0.22%
43	WEILL MEDICAL COLL OF CORNELL UNIV	$771,873.00	$245,178,319.00	0.31%
44	PROVIDENCE PORTLAND MEDICAL CENTER	$769,313.00	$286,778,235.00	0.27%
45	UNIV OF NORTH CAROLINA CHAPEL HILL	$742,620.00	$498,473,940.00	0.15%
46	UNIVERSITY OF OKLAHOMA HLTH SCIENCES CTR	$670,101.00	$67,174,001.00	1.00%
47	UNIV OF ARKANSAS FOR MED SCIS	$623,149.00	$59,468,942.00	1.05%
48	UNIVERSITY OF SOUTHERN CALIFORNIA	$507,076.00	$316,031,543.00	0.16%
49	STATE UNIVERSITY OF NEW YORK AT BUFFALO	$492,918.00	$71,194,190.00	0.69%
50	HENRY FORD HEALTH SYSTEM	$488,310.00	$29,943,791.00	1.63%
51	WEST VIRGINIA UNIVERSITY	$486,459.00	$37,610,206.00	1.29%
52	UNIVERSITY OF MISSOURI-COLUMBIA	$432,877.00	$67,288,728.00	0.64%

53	VIRGINIA COMMONWEALTH UNIVERSITY	$432,387.00	$89,642,815.00	0.48%
54	OREGON HEALTH & SCIENCE UNIVERSITY	$425,651.00	$286,778,235.00	0.15%
55	UNIVERSITY OF ROCHESTER	$400,346.00	$174,075,410.00	0.23%
56	UNIVERSITY OF CALIFORNIA AT DAVIS	$359,138.00	$271,978,114.00	0.13%
57	RBHS-NEW JERSEY MEDICAL SCHOOL	$358,185.00	$50,601,592.00	0.71%
58	UNIVERSITY OF KANSAS MEDICAL CENTER	$352,657.00	$71,611,090.00	0.49%
59	UNIVERSITY OF MIAMI SCHOOL OF MEDICINE	$347,621.00	$154,465,150.00	0.23%
60	UNIVERSITY OF TENNESSEE HEALTH SCI CTR	$298,443.00	$57,934,259.00	0.52%
61	UNIVERSITY OF MINNESOTA	$179,567.00	$336,632,637.00	0.05%

Supplemental table 4: 2021 Funding by institution using the sensitivity analysis' supplementary approach.

Rad Onc Ranking	Institution	2021 Rad Onc Funding Amount	Institution Total 2021 NIH Funding	% of Grant Monies Awarded to Radiation Oncology
1	UNIVERSITY OF TX MD ANDERSON CAN CTR	$10,220,833.00	$172,556,179.00	5.92%
2	STANFORD UNIVERSITY	$7,715,692.00	$611,354,637.00	1.26%
3	COLUMBIA UNIVERSITY HEALTH SCIENCES	$7,434,435.00	$580,097,026.00	1.28%
4	UNIVERSITY OF MICHIGAN AT ANN ARBOR	$5,787,549.00	$609,038,367.00	0.95%
5	UNIVERSITY OF PENNSYLVANIA	$5,646,451.00	$641,789,096.00	0.88%
6	YALE UNIVERSITY	$5,395,337.00	$557,452,201.00	0.97%
7	UNIVERSITY OF WISCONSIN-MADISON	$5,109,034.00	$367,581,113.00	1.39%

8	UNIVERSITY OF CALIFORNIA, SAN FRANCISCO	$5,101,250.00	$709,018,244.00	0.72%
9	WASHINGTON UNIVERSITY	$4,955,508.00	$623,444,643.00	0.79%
10	UT SOUTHWESTERN MEDICAL CENTER	$4,380,721.00	$255,325,647.00	1.72%
11	OHIO STATE UNIVERSITY	$4,202,923.00	$230,407,670.00	1.82%
12	UNIVERSITY OF MARYLAND BALTIMORE	$4,014,903.00	$234,014,725.00	1.72%
13	UNIVERSITY OF CALIFORNIA LOS ANGELES	$3,774,459.00	$590,125,984.00	0.64%
14	EMORY UNIVERSITY	$3,341,029.00	$479,575,357.00	0.70%
15	DUKE UNIVERSITY	$3,328,166.00	$731,237,450.00	0.46%
16	UNIVERSITY OF CALIFORNIA-IRVINE	$3,008,921.00	$172,170,439.00	1.75%
17	JOHNS HOPKINS UNIVERSITY	$2,914,274.00	$824,856,274.00	0.35%
18	UNIVERSITY OF CALIFORNIA, SAN DIEGO	$2,456,097.00	$549,764,120.00	0.45%
19	UNIV OF NORTH CAROLINA CHAPEL HILL	$2,414,878.00	$498,473,940.00	0.48%

20	ICAHN SCHOOL OF MEDICINE AT MOUNT SINAI	$2,197,443.00	$441,276,662.00	0.50%
21	UNIVERSITY OF ALABAMA AT BIRMINGHAM	$2,188,816.00	$327,424,300.00	0.67%
22	MEDICAL COLLEGE OF WISCONSIN	$1,998,741.00	$115,235,521.00	1.73%
23	WEILL MEDICAL COLL OF CORNELL UNIV	$1,858,411.00	$245,178,319.00	0.76%
24	UNIVERSITY OF UTAH	$1,681,625.00	$229,692,858.00	0.73%
25	UNIVERSITY OF IOWA	$1,655,300.00	$183,267,904.00	0.90%
26	UNIVERSITY OF MIAMI SCHOOL OF MEDICINE	$1,621,861.00	$154,465,150.00	1.05%
27	NEW YORK UNIVERSITY SCHOOL OF MEDICINE	$1,588,954.00	$809,311,644.00	0.20%
28	UNIVERSITY OF COLORADO DENVER	$1,533,690.00	$313,670,852.00	0.49%
29	THOMAS JEFFERSON UNIVERSITY	$1,507,933.00	$81,449,194.00	1.85%
30	NORTHWESTERN UNIVERSITY AT CHICAGO	$1,243,830.00	$367,542,487.00	0.34%
31	UNIVERSITY OF CHICAGO	$1,226,034.00	$234,209,363.00	0.52%

32	UNIVERSITY OF PITTSBURGH AT PITTSBURGH	$1,166,361.00	$597,804,833.00	0.20%
33	UNIV OF ARKANSAS FOR MED SCIS	$1,147,800.00	$59,468,942.00	1.93%
34	UNIVERSITY OF TEXAS HLTH SCIENCE CENTER	$1,139,086.00	$95,847,459.00	1.19%
35	UNIVERSITY OF VIRGINIA	$1,019,615.00	$175,512,234.00	0.58%
36	UNIVERSITY OF KANSAS MEDICAL CENTER	$963,760.00	$71,611,090.00	1.35%
37	UNIVERSITY OF WASHINGTON	$883,459.00	$543,508,259.00	0.16%
38	INDIANA UNIV-PURDUE UNIV AT INDIANAPOLIS	$862,338.00	$230,433,043.00	0.37%
39	CASE WESTERN RESERVE UNIVERSITY	$513,265.00	$177,329,462.00	0.29%
40	VIRGINIA COMMONWEALTH UNIVERSITY	$432,387.00	$89,642,815.00	0.48%
41	UNIVERSITY OF ROCHESTER	$400,346.00	$174,075,410.00	0.23%
42	UNIVERSITY OF CALIFORNIA AT DAVIS	$359,138.00	$271,978,114.00	0.13%

43	UNIVERSITY OF NEBRASKA MEDICAL CENTER	$306,167.00	$89,469,340.00	0.34%
44	UNIVERSITY OF FLORIDA	$263,754.00	$212,488,586.00	0.12%

Supplemental figure 2: 2021 NIH RePORTER department categorization breakdown

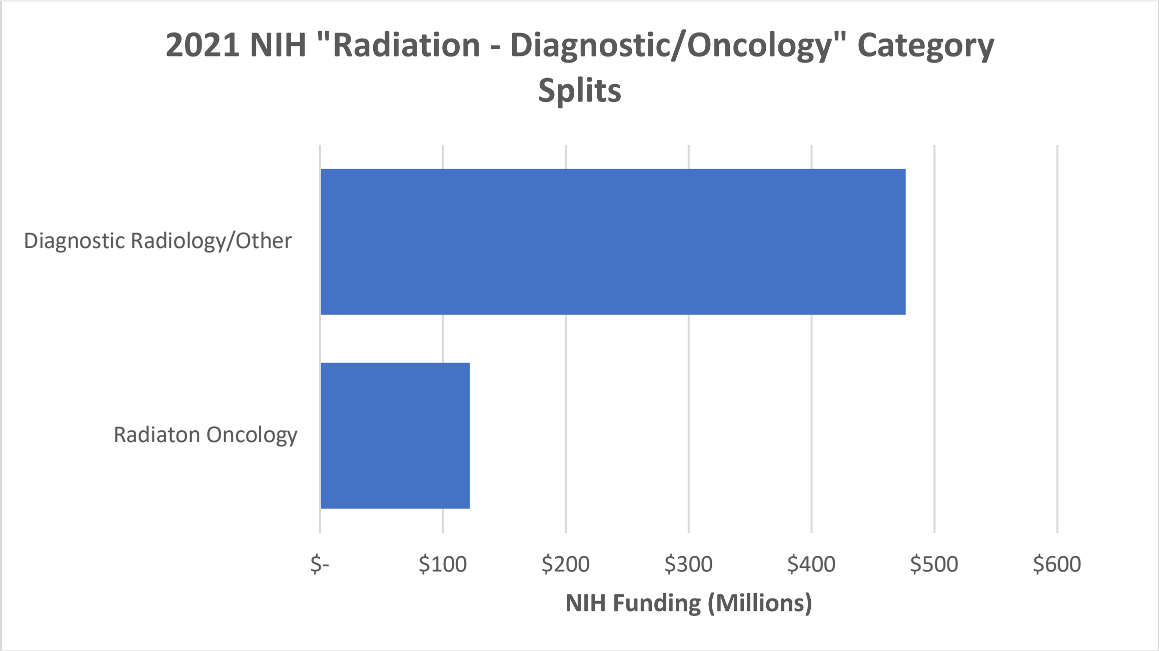

Supplement: Manuscript Supplementary [file mmc1.pdf]
